# Capacitive-Type Pressure-Mapping Sensor for Measuring Bite Force

**DOI:** 10.3390/ijerph19031273

**Published:** 2022-01-24

**Authors:** Masanori Iwasaki, Ichinosuke Maeda, Yota Kokubo, Yoshitomo Tanaka, Tetsuji Ueno, Wataru Takahashi, Yutaka Watanabe, Hirohiko Hirano

**Affiliations:** 1 Tokyo Metropolitan Geriatric Medical Center, 35-2 Sakae-cho, Itabashi-ku, Tokyo 173-0015, Japan; iwasaki@tmig.or.jp (M.I.); wataru.takahashi@jp.sumitomoriko.com (W.T.); ywata@den.hokudai.ac.jp (Y.W.); h-hiro@gd5.so-net.ne.jp (H.H.); 2 Sumitomo Riko Co., Ltd., 3-1 Higashi, Komaki-shi 485-0041, Japan; ichinosuke.maeda@jp.sumitomoriko.com (I.M.); yoshitomo.tanaka@jp.sumitomoriko.com (Y.T.); tetsuji.ueno@jp.sumitomoriko.com (T.U.); 3 Gerontology, Department of Oral Health Science, Faculty of Dental Medicine, Hokkaido University, Kita 14-Jo Nishi 5-Chome, Kita-ku, Sapporo-shi 060-8586, Japan

**Keywords:** bite force, pressure-mapping sensor, oral health

## Abstract

Bite force is an important indicator of masticatory performance. However, existing methods for measuring bite force are either ineffective or expensive. Hence, we developed a novel capacitive-type pressure-mapping sensor that converts mechanical forces into changes in capacitance and calculates bite force. A portable device was fabricated based on this sensor sheet, and the accuracy of the bite-force measurements provided by the device was evaluated. The sensor has a thickness of 1.6 mm and has 63 measurement points. It was inserted into a dental model, where the output value was measured and compared with that of a universal testing machine (AG-IS 100 kN). A regression equation to estimate the bite force was obtained based on the relationship between the output of the capacitive-type pressure-mapping sensor and that of the load cell of the universal testing machine. The estimated bite force from the sensor and the quadratic regression equation closely resembled the known load applied by the compression tester (R^2^ = 0.992). We therefore conclude that the developed sensor can measure bite force accurately and effectively. A device with a built-in capacitive-type pressure-mapping sensor can potentially be a user-friendly tool for bite-force measurements in both clinical and epidemiological settings.

## 1. Introduction

Based on a large-scale, population-based epidemiological study [1], it was seen that bite force, an objective measure of oral function, was associated with masticatory performance regardless of occlusal status. Results of other studies [1,2,3] also suggests that bite force is a key determinant of masticatory performance. Previous studies reported that bite force is associated with dietary intake, cognitive and physical functions, and cardiovascular disease [4,5,6,7,8]. In addition, bite force is one of the diagnostic components of oral hypofunction, a seven-component phenotype of the clinical features of oral health [9]. Therefore, bite-force measurement is considered an important and objective approach for evaluating masticatory performance, as well as a suitable parameter for investigating the link between oral and general health in both clinical and epidemiological settings.

Currently, several systems for bite pressure distribution and/or bite-force measurement are commercially available in Japan. T-Scan (Tekscan, Inc., South Boston, MA, USA) is a system that uses a thin film pressure-mapping sensor sheet to assist dentists in performing occlusal analyses on their patients [10,11]. Although it can evaluate the bite pressure and pressure distribution, it is inaccurate for measuring bite force [12]. Additionally, it is relatively expensive (USD 13,537 for the system and USD 115 for 10 sheets [USD 1 = JPY 109 as of 28 April 2021]) [13]. Another commercially available system is Dental Prescale (GC Corp., Tokyo, Japan), which uses pressure-sensitive sheets [14]. Its installation and operating costs are much cheaper than those of the T-scan system (USD 3898 and USD 272 for 40 sheets) [15]. Furthermore, Dental Prescale can measure both the bite force and pressure distribution. However, to scan the pressure-sensitive sheet that the individuals bites, the bite pressure distribution and bite-force measurements require a special device. Dental Prescale can only map the pressure distribution after clenching in the intercuspal position, and hence cannot evaluate it during clenching. Moreover, if the measurement fails, a new pressure-sensitive sheet must be prepared because sheets cannot be reused. MPX5700 (Motorola, SPS, Austin, TX, USA) is a pneumatic pressure transducer. In this system, pipes and sensors are connected to an analog-to-digital converter. The system has a software for reading the pressure changes [16]. The MPX 5700 pressure sensor media is only suitable for use with air. The accuracy and reliability will be deteriorated with any other pressure medium except for dry air [17]. There are other systems that use strain gauges [18,19] or optical fibers [20], and although highly accurate, they are unsuitable for measurements because they have an enlarged size and mass. The Occlusal Force-Meter GM10 (NAGANO KEIKI Co., Ltd., Tokyo, Japan) consists of a hydraulic pressure gauge with a biting element made of a vinyl material, encased in a polyethylene tube called disposable occlusal cap [12]. Although the accuracy and repeatability of this device were confirmed [21], this device went out of production.

We developed a novel capacitive-type pressure-mapping sensor that consists of thin and flexible rubber/sponge material, enabling the monitoring of bite force and pressure distribution during clenching. This sensor converts mechanical forces into changes in capacitance to calculate bite force. Based on this sensor sheet, a portable device for bite-force measurements was fabricated. This study was designed to evaluate the accuracy and effectiveness of this new bite-force-measuring device by comparing the output value of the capacitive-type pressure-mapping sensor with that of a universal testing machine (AG-IS 100 kN, Shimadzu Co., Ltd., Kyoto, Japan), which can provide accurate force to the test object [22].

## 2. Materials and Methods

### 2.1. Design and Usage of Sensor Sheets

**Figure 1:**
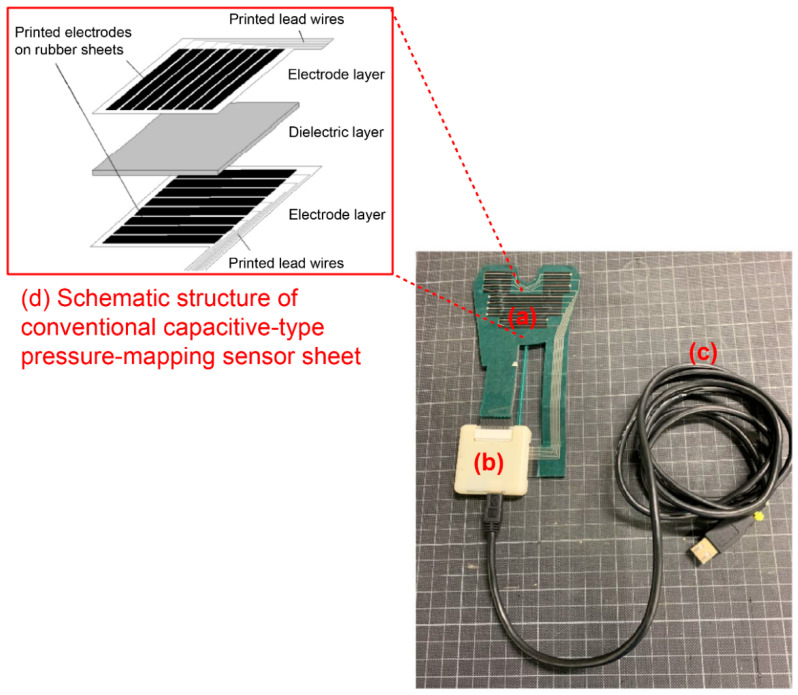
Appearance of capacitive-type pressure-mapping sensor. Figure 1 legend: The capacitive-type pressure-mapping sensor consists of (**a**) a sensor sheet (pressure sensitive area), (**b**) a circuit board for the capacitance value measurement, and (**c**) a USB cable for connecting to a PC. A sensor sheet (**a**) has a three-layer structure, composed of a dielectric layer (urethane sponges) sandwiched between two polymer film sheets (polyethylene terephthalate) as shown in (**d**), and was designed according to the shape of the dental arch. Electrodes were patterned on a polymer film sheet using a screen-printing technique involving special ink. Specifically, polymer-type silver paste was printed for the connection wire, then polymer-type carbon paste was printed for the electrode, and finally, polyester resin-based paste was printed as the protecting layer. Electrodes were arranged such that the electrodes on the two sides intersected at right angles. One capacitive-type sensor was formed by the intersection of two electrodes. The thickness of the sensor sheet was 1.6 mm, which was reduced to 0.3 mm or less when a bite load was applied.

The appearance of the capacitive-type pressure-mapping sensor is shown in Figure 1.

The capacitance of the 63 points on one sensor sheet, formed by the intersection of 7 electrodes on one side of the electrode layer and 9 on the other, can be expressed by the following equation:

C (i, j) = ε_0_ε_r_ (S (i, j)/d(i, j) (i = 1–7; j = 1–9) (1)

Here, C is the capacitance value of the point (i, j), ε_0_ is the permittivity of the vacuum, ε_r_ is the relative permittivity of the dielectric layer, S is the electrode area of the point (i, j), and d is the thickness of the dielectric layer. The value of d depends on the pressure loading at each point. Because S, the electrode area of each point, was constant, C can be varied by the change in d; thickness of the dielectric layer. When a load is applied, the capacitance at each pressure-sensitive point increases as the thickness of the dielectric layer decreases; thus, the capacitance changes owing to the load. By sequentially switching the electrodes on both sides, the capacitance expressed in Equation (1) is determined in a circuit board in the sensor (Figure 1b) using the vector impedance meter method [23].

### 2.2. Comparison of Output Value against Compressive Load

A capacitive-type pressure-mapping sensor was inserted into a dental model (hos-j19, Syom, Southernwind Corp., Nagano, Japan). The maxillary intercanine width (i.e., the linear distance between the cusp tips of the canines) was 33 mm. The maxillary intermolar width (i.e., the linear distance between the mesiobuccal cusp tips of the molars) was 52 mm. The maxillary arch length (i.e., the distance between the line connecting the distal surface of the first molars and the labial surface of the central incisors) was 42 mm. The width of the mandibular intercanine was 26 mm. The mandibular intermolar width (i.e., the distance between the most gingival extensions of the buccal groove on the molars) was 48 mm. The length of the mandibular arch was 40 mm.

**Figure 2:**
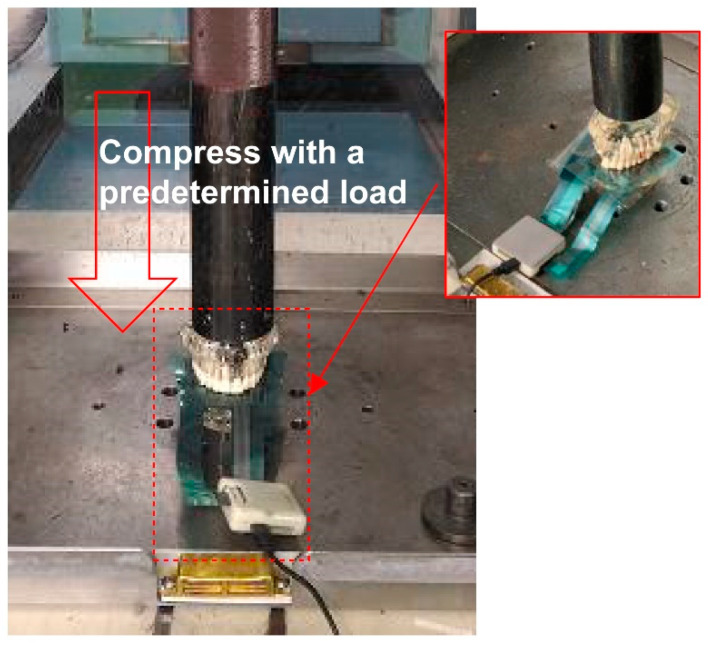
Capacitive-type pressure-mapping sensor installed on the dental model.

A predetermined load was applied to the upper surface of the dental model using a universal testing machine (Figure 2) operating at 25 °C. Various types of strength tests were conducted using this universal testing machine, including tensile, compression, bending, tearing, peeling, creep, and stress relaxation testing. This machine can provide accurate test force to the test object with a measurement accuracy of within ±1.0% of the specified test force [22].

The test involved compression with predetermined loads of 50, 100, 150, 200, 300, 500, and 800 N applied by the universal testing machine at a constant rate of 10 mm/min. One sensor sheet was used through each test with the predetermined load of 50 to 800 N.

The total change in capacitance at each pressure-sensitive point was recorded. The initial capacitance value at each pressure-sensitive point was recorded using the output value of the universal testing machine when there was no load. Because the sensor sheet was made of polymer, stress relaxation could not be completely avoided. Therefore, an average value of the capacitance values measured in 1 s at a cycle of 5 Hz, after reaching the predetermined load, was recorded and used for subsequent bite-force calculation. Using the output value of each pressure-sensitive point of the capacitive-type pressure-mapping sensor, a regression equation for the bite-force calculation was estimated. The goodness-of-fit for the regression models was evaluated using R^2^.

Next, the possibility of the reusability of the sensor sheets was tested. The capacitive-type pressure-mapping sensor was inserted into dental model A, and the test was conducted 10 times in succession without removing the capacitive-type pressure-mapping sensor sheet. The sensor sheet is made of soft material, and hysteresis reduces the accuracy of low-pressure measurements when the sheet is used continuously. Therefore, an interval of approximately 1 min was set between each test. The variability was estimated using the percent coefficient of variance (CV%), which was obtained by multiplying 100 with SD/M, where SD represents the standard deviation, and M the mean value.

Finally, the effects of the differences in the dental models on bite-force measurement were assessed. In addition to the dental model that was used for the above examinations (dental model A), another model (dental model B; OYOJ011442, Oyodental, Chongqing, China) was prepared. The maxillary intercanine of dental model B was measured to be 34 mm, the width of the maxillary intermolar was 52 mm, the length of the maxillary arch was 33 mm, the width of the mandibular intercanine was 26 mm, the mandibular intermolar width was 48 mm, and the length of the mandibular arch was 32 mm. The capacitive-type pressure-mapping sensor was inserted into dental models A and B. The output values were recorded under loads of 50, 100, 150, 200, 300, 500, and 800 N, which were applied using the universal testing machine.

Calculations and statistical analyses were performed using Microsoft Excel (Microsoft Corp., Redmond, WA, USA).

## 3. Results

### 3.1. Comparison of Output Characteristics of Capacitive-Type Pressure-Mapping Sensor and Universal Testing Machine against Compressive Load

**Figure 3:**
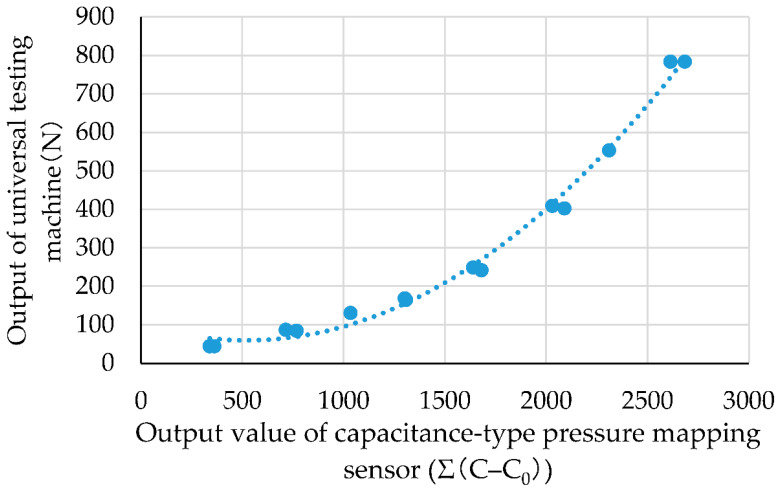
Relationship between sensor output and universal testing machine output.

Figure 3 shows the relationship between the outputs of the sensor and the universal testing machine when the pressure was applied. The horizontal axis represents the total value of the capacitive-type pressure-mapping sensor, and the vertical axis represents the corresponding output value of the load cell of the universal testing machine. The tests were conducted twice under the same conditions. The output values of both the universal testing machine and the sensor sheet were plotted. Table S1 presents raw data of Figure 3. From the result, the quadratic regression equation y = 1.5594 × 10^−4^x^2^ − 1.6205 × 10^−1^x + 1.0149 × 10^2^, R^2^ = 0.991 (where x is the total capacitive-type pressure-mapping sensor output value such that x = Σ (C − C_0_), and y is the output of the universal testing machine) was obtained.

**Figure 4:**
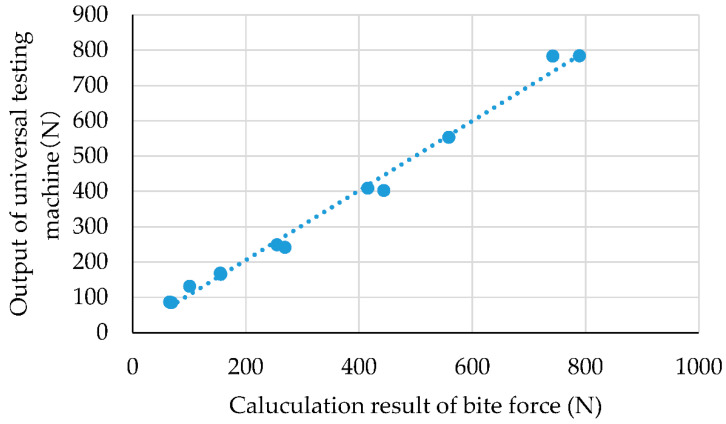
Calculated result of bite force using quadratic regression equation.

Figure 4 shows the calculated results of the bite force from the quadratic regression equation. The horizontal axis represents the calculated result of the bite force from the total value of the capacitive-type pressure-mapping sensor using the quadratic regression equation, and the vertical axis represents the corresponding output value of the load cell of the universal testing machine. Table S2 presents the raw data of Figure 4. For example, when the load cell of the universal testing machine was at 87 N, the total capacitive-type pressure-mapping sensor output was recorded at 714 digits. This value is assigned to x in the quadratic regression equation y = 1.5594 × 10^−4^x^2^ − 1.6205 × 10^−1^x + 1.0149 × 10^2^. As a result, the bite force is calculated to be 65 N. Overall, the estimated bite force was found to agree with the load cell output of the universal testing machine (R^2^ = 0.992).

### 3.2. Characteristics of Bite Force Recorded by Capacitive-Type Pressure-Mapping Sensor

**Figure 5:**
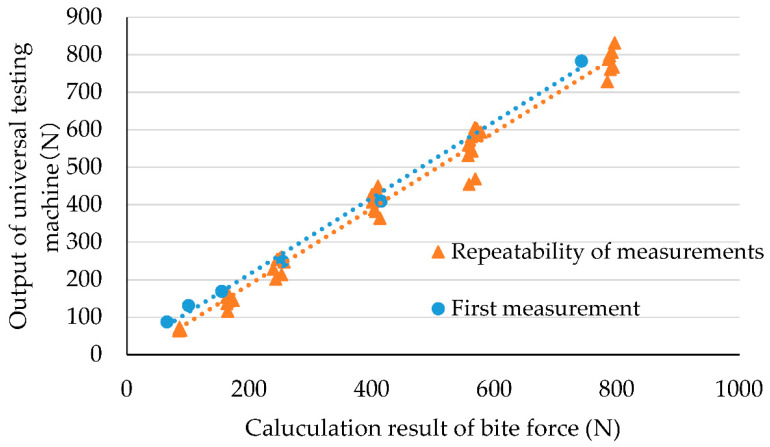
Repeatability tests showing evaluation of changes in output.

Figure 5 shows an evaluation of the repeatability of the results, where 10 repeated measurements under the same conditions were used. The horizontal axis represents the calculated result of the bite force from the total value of the capacitive-type pressure-mapping sensor using the quadratic regression equation, and the vertical axis represents the corresponding output value of the load cell of the universal testing machine. Blue dots represent the first measurement and orange dots represent the subsequent measurements (from the 2nd to the 10th time). The blue dotted line represents the regression line based on the first measurement, and the orange dotted line represents the regression line based on subsequent measurements. Table S3 presents the raw data of Figure 4. M (mean value) and SD were calculated to be 0.93 and 0.11, respectively, resulting in a CV% of 11.8.

**Figure 6:**
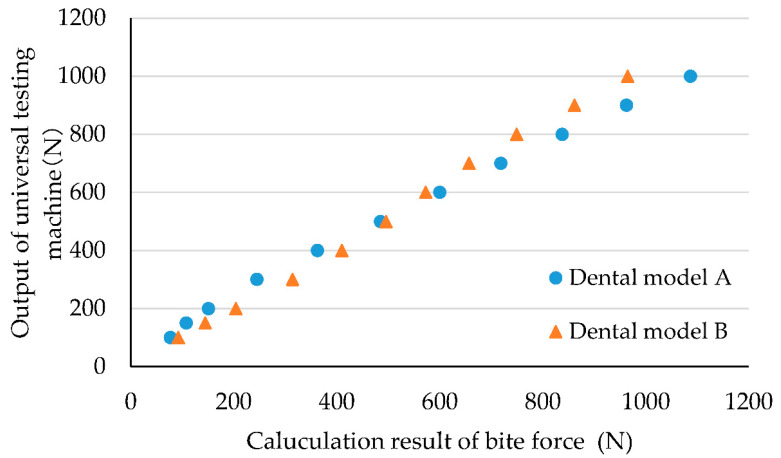
Effect of type of dental model.

Figure 6 shows the correlation of the bite force obtained with a capacitive-type pressure-mapping sensor and the force precisely generated by the universal testing machine according to the type of dental model. The horizontal axis represents the calculated result of the bite force from the total value of the capacitive-type pressure-mapping sensor using the quadratic regression equation, and the vertical axis represents the corresponding output value of the load cell of the universal testing machine. Blue dots represent the values from the measurement using dental model A, and the orange dots represent the values from the dental model B. Table S4 presents the raw data of Figure 6. M (mean value) and SD were calculated to be 0.95 and 0.10, respectively, resulting in a CV% of 10.5.

## 4. Discussion

In this study, we developed a new capacitive-type pressure-mapping sensor to measure bite force. A novel portable device for bite-force measurements was fabricated based on a capacitive-type sensor sheet. We obtained a quadratic regression equation to estimate the bite force based on the relationship between the output value of the capacitive-type pressure-mapping sensor and the output value of the load cell of the universal testing machine. The model fit was excellent (R^2^ = 0.991). Furthermore, the bite force estimated from the capacitive-type pressure-mapping sensor using the regression equation showed a close resemblance to the load applied by the compression tester (R^2^ = 0.992).

The newly developed sensor sheet is made of thin and flexible rubber/sponge material which contributes to the efficient measurement of the bite force. A new portable device for bite-force measurement enables clinicians and researchers to confirm the bite force immediately without using other equipment, such as a scanner. The results of the repeatability test for the sensor sheet indicated that the sensor sheets were reusable. The effect of using different dental models on the estimated bite force was minimal. In this study, we found that the bite force obtained using a correction formula, given the total value of the changes in capacitance at each pressure-sensitive point, was highly correlated with the output value of the load cell of the universal testing machine. This indicates that there is no need to establish a correction formula at each pressure-sensitive point. Creating a bite-force estimation formula at each point complicates the microcomputer calculation and requires expensive measuring instruments with high precision to calculate the calibration formula during manufacturing, which is a major disadvantage in terms of price. Creating a simple formula for calculating the bite force from the total value of the changes in capacitance at each pressure-sensitive point is an important aspect to consider in terms of cost. Furthermore, this sensor was designed so that there was a slightly decreased resolution to reduce the area between the wires and perform surface-based measurements for the dentition to calculate the bite force, thus ensuring a striking balance between price and performance (degree of accuracy).

Although the sensor sheet is designed to be 0.3 mm or thinner when a bite load is applied, it is thicker than the Dental Prescale system, which is approximately 0.1 mm thick. Notably, our sensor sheet measures the capacitance value when a very soft dielectric (sponge) is compressed by the input pressure. It does not perform measurements when the sensor is separated, i.e., the layers are not in contact. This sensor uses a different mechanism than the Dental Prescale system to measure bite force. Bite-force measurements obtained with this sensor accounted for the occlusal proximity area. Overall, the effect of thickness on the bite-force calculation is reduced when compared to that of the Dental Prescale system. Comparing the performance of this device with other commercially available devices is beyond the scope of this study. Nonetheless, we recognize that comparing the performance of this device with other commercially available systems is an important next step. Furthermore, to assess the significance of the bite-force value, exploring the relationship of bite force measured by the developed sensor and device with other oral functions, including masticatory performance, tongue pressure, and articulatory oral motor skill, as well as physical functions, such as grip strength, is warranted. Based on these results, the device would be revisited and subjected to further deliberation.

## 5. Conclusions

The current study demonstrated that the newly developed capacitive-type pressure-mapping sensor is highly accurate and effective for measuring bite force. A device with built-in capacitive-type pressure-mapping sensors has the potential to be a user-friendly tool for bite-force measurements in clinical and epidemiological settings.

## Data Availability

The data presented in this study are available in Supplementary Materials.

## Supplementary Materials

The following supporting information can be downloaded at https://www.mdpi.com/article/10.3390/ijerph19031273/s1, Table S1: (Raw data) Relationship between sensor output and universal testing machine output, Table S2: (Raw data) Calculated result of bite force using quadratic regression equation, Table S3: (Raw data) Repeatability tests showing evaluation of changes in output, Table S4: (Raw data) Effect of type of dental model.
