# Supplementary material for: Capacitive-Type Pressure-Mapping Sensor for Measuring Bite Force
Source: Int J Environ Res Public Health. 2022 Jan 24;19(3):1273. doi: 10.3390/ijerph19031273 (PMC8834865; doi:10.3390/ijerph19031273)
Supplement: Supplementary file 1 [file ijerph-19-01273-s001.zip › ijerph-1500643-supplementary-2.pdf]

Table S1. (Raw data) Relationship between sensor output and universal testing machine output

Output of the load cell of the universal testing machine (N)	Total value of the capacitive-type pressure-mapping sensor (digit)
First measurement	
44	339
87	714
131	1034
169	1302
249	1639
409	2030
784	2612
Second measurement	
44	339
85	714
165	1034
241	1302
403	1639
553	2030
784	2612

Table S2. (Raw data) Calculated result of bite force using quadratic regression equation

Output of the load cell of the universal testing machine (N)	Total value of the capacitive-type pressure-mapping sensor (digit)	Calculated bite force from the total value of the capacitive-type pressure-mapping sensor using the quadratic regression equation (N)
87	714	65
131	1034	101
169	1302	155
249	1639	255
409	2030	415
784	2612	742
85	767	69
165	1307	156
241	1680	269
403	2089	443
553	2309	559
784	2683	789

Table S3. (Raw data) Repeatability tests showing evaluation of changes in output

A: Output of the load cell of the universal testing machine (N)	Total value of the capacitive-type pressure-mapping sensor (digit)	B: Calculated bite force from the total value of the capacitive-type pressure-mapping sensor using the quadratic regression equation (N)	X=B/A
First measurement			
86	756	68	0.79
166	1251	143	0.86
249	1643	256	1.03
401	2013	407	1.01
557	2258	531	0.95
790	2641	761	0.96
85	766	69	0.81
167	1316	158	0.95
245	1634	253	1.03
406	2057	428	1.05
559	2111	454	0.81
786	2680	787	1.00
Subsequent measurements			
86	756	68	0.79
253	1512	213	0.84
413	1918	364	0.88
569	2140	469	0.82
85	767	69	0.81
168	1291	152	0.90
252	1655	260	1.03
578	2370	593	1.03
85	685	64	0.75
165	1120	116	0.70
243	1473	201	0.83

406	1957	382	0.94
564	2280	543	0.96
86	764	69	0.80
163	1223	137	0.84
239	1559	228	0.95
401	2056	427	1.06
569	2356	585	1.03
792	2708	806	1.02
86	810	73	0.85
163	1293	153	0.94
410	2101	449	1.10
571	2388	604	1.06
796	2745	832	1.05
86	749	68	0.79
250	1637	254	1.02
407	2083	441	1.08
568	2390	605	1.07
785	2590	728	0.93
88	706	65	0.74
174	1259	145	0.83
257	1616	247	0.96
409	2026	413	1.01
558	2310	559	1.00
794	2649	766	0.96
87	771	69	0.79
168	1290	152	0.90
246	1637	254	1.03
404	1969	387	0.96
562	2349	581	1.03
788	2694	797	1.01
		M	0.93
		SD	0.11
		CV%	11.8%

CV = coefficient of variance, M = mean of X, SD = standard deviation of X

Table S4. (Raw data) Effect of type of dental model

A: Output of the load cell of the universal testing machine (N)	B: Calculated bite force from the total value of the capacitive-type pressure-mapping sensor using the quadratic regression equation (N)	X=B/A
Dental model A		
100	77	0.77
150	108	0.72
200	151	0.76
300	245	0.82
400	362	0.91
500	485	0.97
600	600	1.00
700	719	1.03
800	838	1.05
900	963	1.07
1000	1087	1.09
Dental model B		
92	100	0.92
144	150	0.96
204	200	1.02
314	300	1.05
410	400	1.03
496	500	0.99
573	600	0.96
657	700	0.94
749	800	0.94
861	900	0.96
965	1000	0.97
	M	0.95
	SD	0.10
	CV%	10.5%

CV = coefficient of variance, M = mean of X, SD = standard deviation of X
